# Induction of Apoptosis and Inhibition of Epithelial Mesenchymal Transition by *α*-Mangostin in MG-63 Cell Lines

**DOI:** 10.1155/2018/3985082

**Published:** 2018-04-26

**Authors:** Sung-Jin Park, Bong-Soo Park, Su-Bin Yu, Hae-Mi Kang, Hye-Jin Kim, In-Ryoung Kim

**Affiliations:** ^1^Department of Oral Anatomy, School of Dentistry, Pusan National University, Busandaehak-ro 49, Mulguem-eup, Yangsan-si, Gyeongsangnam-do 50612, Republic of Korea; ^2^BK21 PLUS Project, School of Dentistry, Pusan National University, Busandaehak-ro 49, Mulguem-eup, Yangsan-si, Gyeongsangnam-do 50612, Republic of Korea; ^3^Department of Dental Hygiene, Dongeui University, Gaya 1-dong, Busanjin-gu, Busan 47230, Republic of Korea

## Abstract

Osteosarcoma is the most common bone primary malignant tumor and nearly 30% of patients still die from osteosarcoma due to metastasis or recurrence. Thus, it is necessary to develop effective new chemotherapeutic agents for osteosarcoma treatment. *α*-Mangostin is a xanthone derivative shown to have antioxidant and anticarcinogen properties. However, the molecular mechanisms underlying the antimetastatic effects of osteosarcoma remain unclear. In metastasis progression, epithelial mesenchymal transition (EMT) is a process that plays important roles in development, cell polarity, and increased invasion and migration. This study focused on the induction of apoptosis and inhibition of EMT process by *α*-mangostin in human osteosarcoma cell line MG63. *α*-Mangostin treatments on MG63 cells not only showed the several lines of evidence of apoptotic cell death but also inhibited cell migration, invasion, and EMT-inducing transcription factor. In conclusion, we demonstrate that the *α*-mangostin induces apoptosis via mitochondrial pathway and suppresses metastasis of osteosarcoma cells by inhibiting EMT.

## 1. Introduction

Osteosarcoma, which is highly malignant and the most common bone tumor, predominates among teenagers. Although its prevalence is low relative to other cancers, it is fatal if not discovered early [1]. It has several characteristics, including a rapid cell growth rate, high metastatic potential, and local infiltration to other organs [2, 3]. Despite various treatments such as surgery, radiotherapy, and chemotherapy, the five-year survival rates of osteosarcoma patients are still problematic [4]. Due to local invasion, lymphatic spreading, and metastasis, the cancer prognosis is usually poor. Regarding the management of osteosarcoma, the primary problem is the inability to control local metastasis [5]. Hence, it is necessary to find effective new therapeutic agents without side effects. In recent years, natural substances for the prevention and management of cancers have received much attention due to their limited toxicity and multiple bioactivities [6].

The purple mangosteen* (Garcinia mangostana)* is a tropical tree native to Asian countries such as India, the Philippines, Malaysia, and Thailand [7]. The mangosteen pericarp, used as a traditional medicine for many diseases, contains many compounds known as xanthones [8, 9]. Xanthones are associated with a variety of biological activities: cardioprotective [10], anti-inflammatory [11, 12], antibacterial [13], and antioxidant [14]. *α*-Mangostin, one of the xanthones, has demonstrated anticancer activity against various cancer cell lines [7, 15–18]. In our previous studies, *α*-mangostin induced significant cell death through apoptosis and cell cycle arrest in oral squamous cell carcinoma cell lines [19]. However, the effects of *α*-mangostin against osteosarcoma cells are completely unknown.

During cancer progression and metastasis, a cell morphologic conversion process occurs known as an epithelial mesenchymal transition, which is a significant action. EMT is a cellular process that allows polarized epithelial cells, which interact with basal surface membranes, to transform nonpolarized and invasive mesenchymal cells [20]. EMT is characterized by cell adhesion loss, down-regulation of E-cadherin (epithelial molecule), and upregulation of N-cadherin (mesenchymal molecules) [21, 22].

The current study was designed to elucidate the effect of *α*-mangostin on MG-63 osteosarcoma cell apoptosis, EMT, and the molecular mechanism.

## 2. Materials and Methods

### 2.1. Reagents

The following reagents were obtained commercially. *α*-Mangostin was purchased from Chromadex (Irvine, CA, USA). Fetal bovine serum (FBS) and Dulbecco Modified Eagle Medium (DMEM) were obtained from Thermo Fisher Scientific (Pittsburgh, PA, USA). 3-[4,5-Dimethylthiazol-2-yl]2,5-diphenyl tetrazolium bromide (MTT) and dimethyl sulfoxide (DMSO) were purchased from DUCHEFA Biochem (Haarlem, The Netherlands). Insert-type transwell permeable supports were obtained from Corning (Kennebunk, ME, USA). Antibodies against caspase-3, cleaved caspase-3, PARP, E-cadherin, slug, snail, ERK, phospho ERK, JNK, phosphor JNK, p38, and phosphor p38 were purchased from Cell Signaling Technology (Beverly, MA, USA). Bcl-2, Bak, caspase-9, caspase-7, N-cadherin, and *β*-actin were purchased from Santa Cruz Biotechnology (Santa Cruz, CA, USA). Mouse anti-actin antibody, mouse anti-rabbit IgG antibody, and rabbit anti-mouse IgG antibodies were obtained from Enzo Biochem (Farmingdale, NY, USA).

### 2.2. Cell Culture and *α*-Mangostin Treatment

The human osteosarcoma cell line MG-63 was purchased from ATCC (Rockville, MD, USA). Cells were maintained at 37°C in a humidified atmosphere containing 5% CO_2_ in DMEM supplemented with 10% FBS and 1% penicillin-streptomycin (GIBCO-BRL, Rockville, MD, USA). *α*-Mangostin was dissolved in dimethyl sulfoxide (DMSO) at a stock solution of 20 mM and was kept frozen at −20°C until use. The stock was diluted to their concentration with DMEM when needed. Prior to *α*-mangostin treatment, cells were grown to about 70%–80% confluence and then exposed to *α*-mangostin at different concentrations (0–50 *μ*M) for 24–72 h. Cells grown in a medium containing an equivalent amount of DMSO without *α*-mangostin served as controls.

### 2.3. Cell Cytotoxicity Assay

The cell viability of *α*-mangostin was assessed using an MTT assay. MG-63 cells were seeded in a 96-well plate and treated with *α*-mangostin at various concentrations (0–40 *μ*M) for 24–72 h. And then the cells were treated with MTT stock solution and incubated at 37°C with 5% CO_2_ for 4 h. After that, the MTT solution was removed and DMSO was added to each well. The absorbance was recorded on a microplate reader (Tecan, Männedorf, Switzerland) at a wavelength of 620 nm.

### 2.4. Hoechst Staining

MG-63 cells were seeded in a 60 mm culture dish at a density of 3 × 10^5^ cells/dish and treated with 10–50 *μ*M *α*-mangostin for 24 h. The cells were washed with phosphate buffered saline (PBS) and fixed in a 4% paraformaldehyde (PFA) solution for 10 min. After fixation, the cells were washed again and stained with 1 *μ*g/ml Hoechst 33342 for 10 min at 37°C chamber. Finally, the slides were mounted with glycerol. Each sample was observed under an epifluorescence microscope (Axioskop, Carl Zeiss, Göttingen, Germany).

### 2.5. Flow Cytometry

MG-63 cells were seeded in a 60 mm culture dish at a density of 3 × 10^5^ cells/dish and treated with 10–50 *μ*M *α*-mangostin for 24 h. The harvested cells were washed with PBS containing 1% bovine serum albumin and fixed 95% ethanol with 0.5% Tween 20 to a final concentration of 70% ethanol. The cells were then stained with 10 *μ*g/ml propidium iodide (PI) in 1 ml PBS containing 50 *μ*g/ml RNase A (Invitrogen, Carlsbad, CA, USA) at 4°C for 30 min. Finally, the cells were measured using a CYTOMICS FC500 flow cytometry system (Beckman Coulter, FL, CA, USA). MG-63 cells were seeded in a 60 mm culture dish at a density of 3 × 10^5^ cells/dish. After treatment, the cells were washed with phosphate buffered saline (PBS) and 1 mg/ml of JC-1 was added directly to the cell culture medium. The analysis of MMP was measured on a CYTOMICS FC500 flow cytometry system (Beckman Coulter, FL, CA, USA). Data was analyzed using CXP software, version 2.2.

### 2.6. Invasion and Migration Assay

A cell invasion assay (transwell assay) and a migration assay were conducted to examine the capacity of cell invasion and migration, as described previously. A transwell with an 8.0 *μ*m pore polycarbonate membrane (Corning Costar, Cambridge, MA, USA) was coated with 40 *μ*l Matrigel at 200 *μ*g/ml and incubated for 2 h. Next, MG-63 cells were seeded and treated with 10–25 *μ*M *α*-mangostin. The upper chamber of the transwell was filled with serum-free medium and the lower chamber with 800 *μ*l medium containing 10% FBS for 48 h of incubation at 37°C in a humidified 5% CO_2_ atmosphere. The cells were fixed in methanol and stained with hematoxylin for 30 min and were then counted under an inverted microscope (Olympus, Tokyo, Japan).

### 2.7. Western Blot Analysis

MG-63 cells were lysed in RIPA buffer (Cell Signaling; Danvers, MA) at 4°C for 1 h, which includes pH 7.6 50 mM Tris-Cl, 300 mM NaCl, 0.5% Triton X-100, 2 *μ*l/ml aprotinin, 2 mM PMSF, and 2 *μ*l/ml leupeptin. Total cellular proteins (25–30 *μ*g) in the cell lysate were separated by 10% SDS polyacrylamide gel electrophoresis and the proteins on the gel were then transferred onto a membrane. The membranes were blocked with skim milk blocking buffer for 2 h at room temperature. After blocking, the membranes were kept in a 4°C refrigerator for 24 h with the respective primary antibodies. The next day the membranes were treated with secondary antibodies for 2 h at room temperature. Protein bands were exposed to Alpha Imager HP (Alpha Innotech, Santa Clara, CA, USA). Bcl-2, Bak, caspase-9, 7, 3, cleaved caspase-3, PARP, E-cadherin, N-cadherin, slug, snail, and *β*-actin were used as the primary antibodies, while anti-rabbit and anti-mouse antibodies were used as the secondary antibodies.

### 2.8. Real-Time RT-PCR

Gene expression was analyzed with real-time reverse transcription-polymerase chain reaction (RT-PCR). Total RNA was isolated from MG-63 cells using a RNeasy Mini Kit (Qiagen, Hilden, DEU). Total RNA concentration was determined with a NanoDrop 2.0 spectrophotometer (Thermo Fisher Scientific, Pittsburgh, PA, USA), and 2 *μ*g total RNA was reversibly transcribed into cDNA for 5 min at 70°C using a M-MLV cDNA Synthesis Kit (Enzynomics, Dajeon, KOR). PCR was performed using the TOPreal™ qPCR 2X PreMIX (Enzynomics, Dajeon, KOR) and run on an ABI 7500 Fast Real-Time PCR System (Applied Biosystems 7500 System, Foster City, CA, USA; using Sequence Detection System software version 2.0.1). The relative mRNA levels were normalized using GAPDH as a housekeeping gene.

### 2.9. Statistical Analysis

Reported data represent mean ± SE. Statistical significance between groups was analyzed using one-way analysis of variance (ANOVA) and Dunnett's comparison. Differences with probability (*p*) values < 0.05 were considered statistically significant.

## 3. Results

### 3.1. *α*-Mangostin Affected Cell Viability and Induce Apoptosis in MG-63 Cells

**Figure 1:**
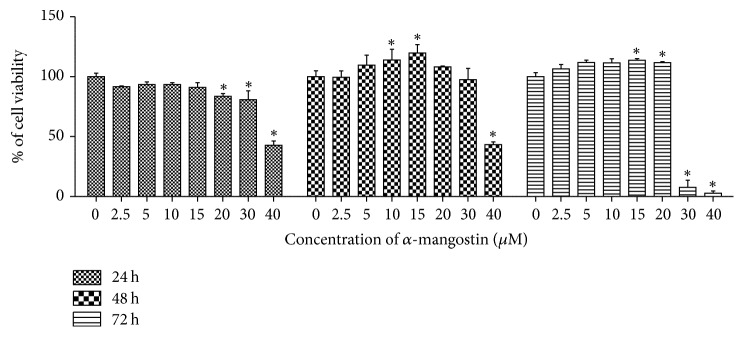
Cell viability assay was performed to confirm the sensitivity of MG-63 cells to various concentrations of *α*-mangostin (0–40 *μ*M). The data were calculated as percent of vehicle control and expressed as the mean of at least three experiments. The bars represent the mean values ± SD (standard deviation). ^*∗*^*p* < 0.05 versus untreated cells.

**Figure 2:**
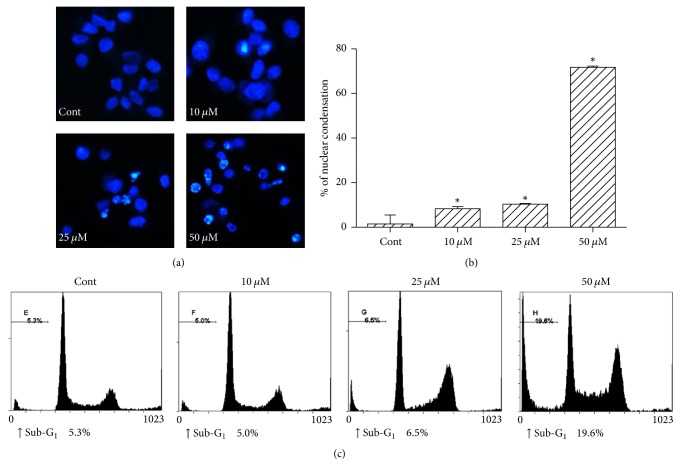
*α*-Mangostin induced apoptosis in MG-63 cells. Cells were treated with 0, 10, 25, and 50 *μ*M *α*-mangostin for 24 h, (a) nuclei were stained with Hoechst staining and observed using a fluorescence microscope. (b) The graph is represented quantification of condensed nuclei. (c) The ratio of sub-G_1_ proportion was determined by FACS analysis. ^*∗*^*p* < 0.05 versus untreated cells.

MG-63 cells were incubated with *α*-mangostin (0–40 *μ*M) for 24, 48, and 72 h and cell viability was assessed with an MTT assay. The result showed that *α*-mangostin decreased the viability of MG-63 cells with the best concentration of cell death at 40 *μ*M (Figure 1). Whether *α*-mangostin-induced cell death is associated with apoptosis, we investigated nuclear condensation and an increase in the sub-G_1_ ratio in the cell cycle, which are hallmarks of apoptosis. Cells were treated with the indicated concentration of *α*-mangostin for 24 h, and Hoechst staining was performed. As shown in Figures 2(a) and 2(b), nontreated group cells showed typically round nuclei, whereas, *α*-mangostin-treated group cells showed condensed nuclei in a dose-dependent manner. To investigate the proportion of sub-G_1_ fraction in MG-63 cells, PI (propidium iodide) staining and flow cytometry analysis were performed. Compared to the control group, *α*-mangostin-treated cells showed an increase in PI positive cells (Figure 2(c)). These results indicated that *α*-mangostin induced apoptosis of MG-63 cells significantly 50 *μ*M *α*-mangostin for 24 h.

### 3.2. *α*-Mangostin-Induced Apoptosis via Mitochondrial Pathway in MG-63 Cells

**Figure 3:**
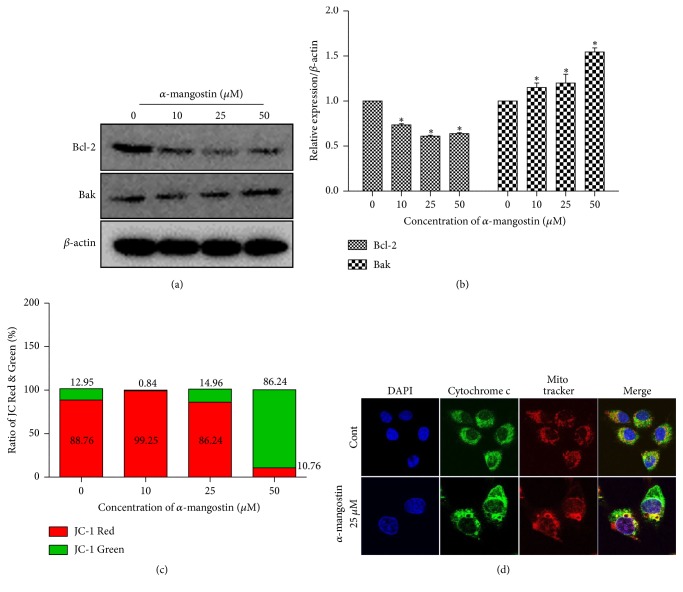
Mitochondrial membrane permeability was increased by *α*-mangostin treatment in MG-63 cells. Cells were treated with *α*-mangostin (0, 10, 25, and 50 *μ*M) for 24 h. (a and b) Bcl-2 and Bak protein expressions exposed with *α*-mangostin were examined by western blot. The *β*-actin is a loading control. (c) MMP reduction was measured by JC-1 staining using FACS analysis. (d) Translocation of cytochrome c from mitochondria into nucleus was observed by a laser confocal fluorescence microscope. ^*∗*^*p* < 0.05 versus untreated cells (control).

**Figure 4:**
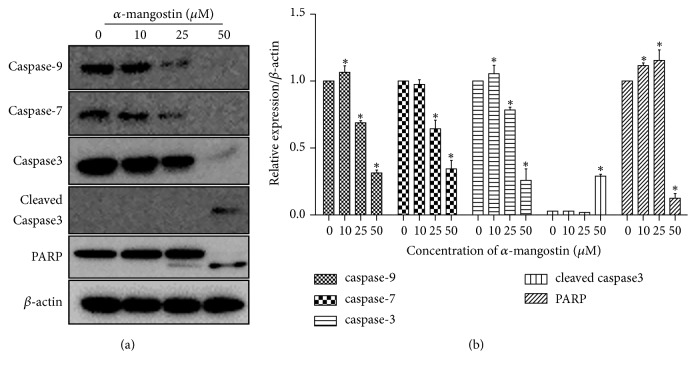
*α*-Mangostin stimulated caspase cascade associated with mitochondrial function. Cells were treated with various concentration of *α*-mangostin (0, 10, 25, and 50 *μ*M) for 24 h. (a) Procaspase-9, procaspase-7, procaspase-3, cleaved caspase-3, and PARP proteins expressions were detected by western blot analysis. The *β*-actin was used as an internal standard. (b) Quantification of proteins expressions was performed by image J. ^*∗*^*p* < 0.05 versus untreated cells (control).

The mitochondrial pathway of apoptosis induced mitochondrial dysfunction through mitochondrial membrane potential (△Ψ*m*) loss [23, 24]. To investigate the effects of *α*-mangostin on mitochondria, we examined various features related to mitochondrial activity after *α*-mangostin treatment for 24 h. Bcl-2 family proteins are associated with mitochondrial membrane; we investigated the expression of Bcl-2 and Bak. The expression level of Bcl-2 decreased, whereas Bak expression increased in a dose-dependent manner (Figure 3(a)). To evaluate the MMP of MG-63 cells were stained with JC-1 and labeled green or red fluorescence. The relative ratio of green to red fluorescence was significantly increased in experimental groups, suggesting that *α*-mangostin caused MMP in MG-63 cells to collapse (Figure 3(b)). The loss of MMT results in the translocation of cytochrome c from mitochondria into cytosol [25]. As shown in Figure 3(c), treatment with *α*-mangostin 25 *μ*M significantly increased the release of cytochrome c into the cytosol. Also, caspase is well known as a key protein in the mitochondrial pathway of apoptosis. The protein expression of caspase-9, -7, -3, and PARP was investigated by western blot. The procaspase-9, -7, and -3 and PARP were downregulated dose-dependently, while on the other hand, cleaved caspase-3 and cleaved PARP were strongly upregulated at 50 *μ*M *α*-mangostin (Figure 4). This data suggested that *α*-mangostin-induced apoptosis is triggered by mitochondrial dysfunction and caspase cascade activation.

### 3.3. *α*-Mangostin Inhibited Invasion and Migration through Suppression EMT in MG-63 Cells

**Figure 5:**
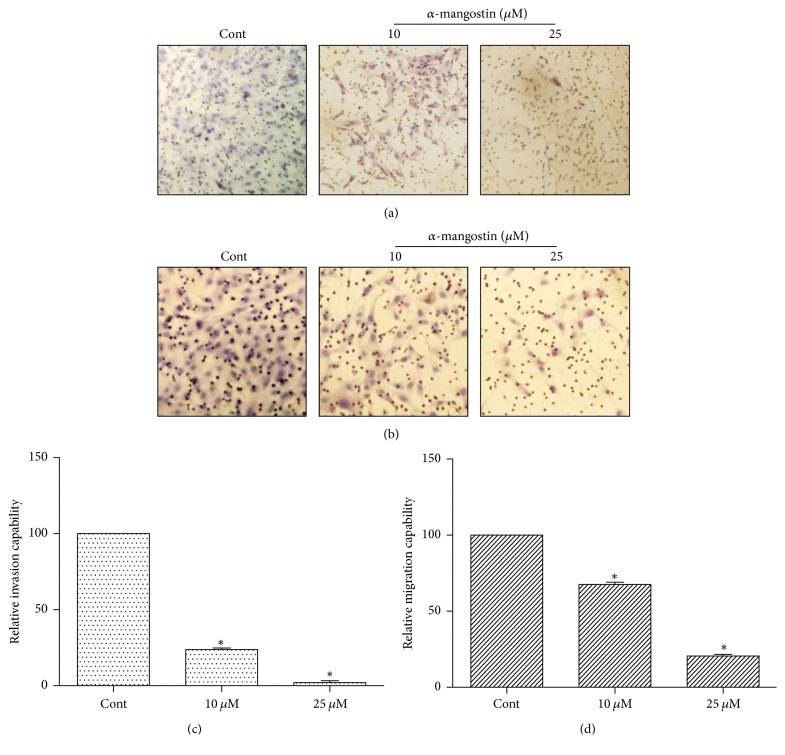
MG-63 cells migration and invasion capability were inhibited by *α*-mangostin. Cells were treated with 10 and 25 *μ*M *α*-mangostin and incubated in transwell for 48 h. (a) Migration and (b) invasion were observed under light microscopy. (c, d) The quantitative analysis was suggested as migration and invasion capability in *α*-mangostin-treated group relative to control. ^*∗*^*p* < 0.05 versus untreated cells.

**Figure 6:**
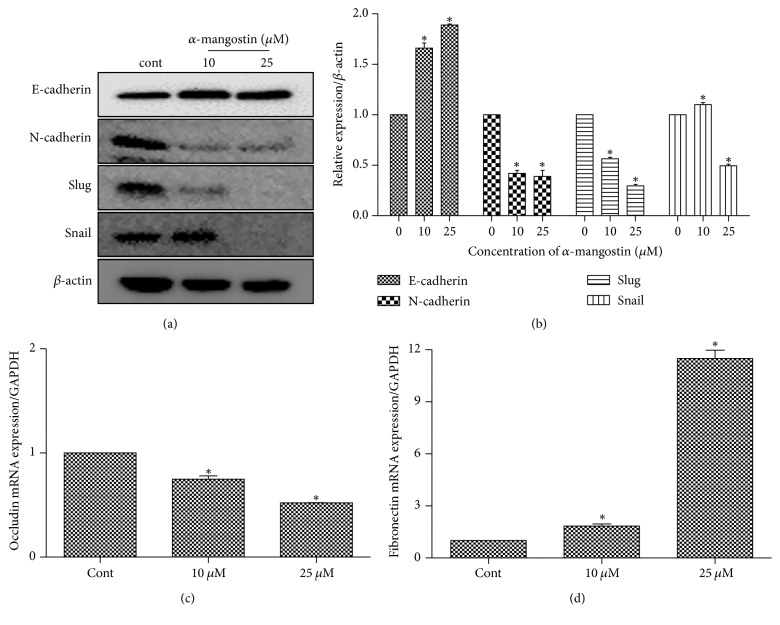
*α*-Mangostin affected EMT-associated marker. Cells were treated with *α*-mangostin (0, 10, and 25 *μ*M) for 48 h. (a) Protein expressions of E-cadherin, N-cadherin, slug, and snail were detected by western blot analysis. (b) Relative protein expressions of EMT markers were determined by image J. (c, d) Occludin, fibronectin expressions in the cells treated with *α*-mangostin (0, 10, and 25 *μ*M) for 48 h were investigated by real-time RT-PCR. ^*∗*^*p* < 0.05 versus untreated cells (control).

Inhibition of cell migration and invasion prevents cancer metastasis into other organs [26]. To elucidate the effect of *α*-mangostin on MG-63 EMT, transwell migration and invasion assay were performed. Cells were exposed to *α*-mangostin (0, 10, and 25 *μ*M) for 48 h; we observed a significant reduction in the migratory capacity of cells compared to the control (Figures 5(a) and 5(c)). Moreover, the invasive capability of MG63 cells was also suppressed by *α*-mangostin treatment (Figures 5(b) and 5(d)). In addition, EMT was closely associated with tumor migration and invasion, to evaluate the effects of *α*-mangostin on EMT-related marker protein expression such as E-cadherin, N-cadherin, slug, and snail. Western blot data showed that *α*-mangostin significantly increased E-cadherin, which is an epithelial marker, whereas mesenchymal markers, including N-cadherin, slug, and snail, decreased in a dose-dependent manner (Figures 6(a) and 6(b)). To further characterize the mRNA expression level of EMT-related gene, we examined the real-time RT-PCR. The result showed that *α*-mangostin decreased the mRNA expression of occludin and increased the mRNA expression of fibronectin (Figures 6(c) and 6(d)). These results implied that *α*-mangostin inhibited MG-63 cell migration and invasion through the upregulation of epithelial markers and downregulation of mesenchymal markers.

### 3.4. *α*-Mangostin Suppressed the Phosphorylation of MAPK Pathways in MG-63 Cells

**Figure 7:**
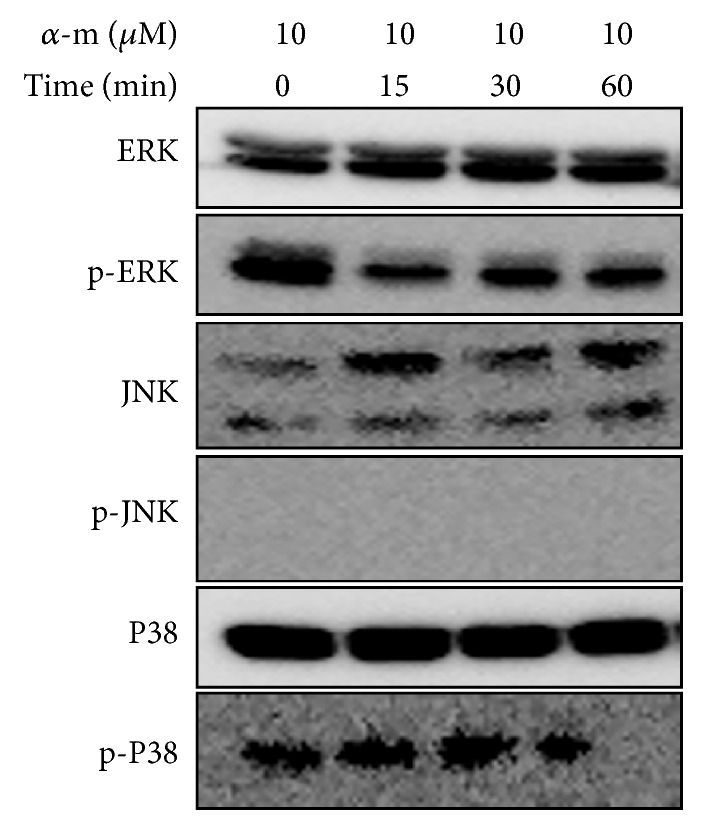
*α*-Mangostin suppressed MAPK signaling phosphorylation. Cells were treated with 10 *μ*M of *α*-mangostin for indicated time-period. The expression levels of MAPK members (ERK, JNK, and p38) were confirmed by western blot assay.

Members of the mitogen-activated protein kinase (MAPK) family consist of ERK1/2, JNK, and p38 and play important roles in cell apoptosis and migration [27]. Thus, in order to investigate whether *α*-mangostin-induced apoptosis and EMT inhibition are associated with MAPK pathways, we verified the protein expressions of MAPK signaling. As shown in Figure 7, phosphorylation of ERK1/2 and p38 expressions were downregulated by 10 *μ*M *α*-mangostin in a time-dependent manner. However, the phosphorylation form of JNK was not detected by western blot. This data demonstrated that 10 *μ*M of *α*-mangostin, which is a concentration of EMT suppression, inhibited MAPK, signaling activation.

## 4. Discussion

The pericarp of mangosteen is an abundant source of polyphenolic compounds, including xanthones. There are three types of xanthone derivatives: *α*-mangostin, *β*-mangostin, and *γ*-mangostin; out of these, *α*-mangostin has been recognized as the most potent xanthone and, as a result, has attracted the most interest due to its properties [7, 28]. In our previous study, *α*-mangostin was demonstrated to have a potent anticancer effect in human oral squamous cell carcinoma cell lines (OSCC) through apoptosis and cell cycle arrest [19]. Also, *α*-mangostin has been reported to inhibit migration and invasion in various cancer cells, including melanoma cells, pancreatic cancer cells, and lung adenocarcinoma cells [17, 29–31]. However, the effect of *α*-mangostin on human osteosarcoma cells is uncertain. Therefore, our study investigated the anticancer effects of *α*-mangostin on human osteosarcoma MG-63 cells. Induction of apoptosis is a well-known regulation method in cancer treatment. Apoptosis is a type I programmed cell death characterized by cell shrinkage, nuclear condensation, and accumulation of sub-G_1_ [32]. In our study, we found that *α*-mangostin induced nuclear condensation and an increase in the proportion of sub-G_1_. The intrinsic pathway of apoptosis is mediated with mitochondria membrane permeability loss caused by the destruction of the outer mitochondrial membrane and the release of proapoptotic proteins [33]. Bcl-2 family proteins are critical controllers of the mitochondria; these proteins are divided into antiapoptotic proteins and proapoptotic proteins [34]. Bcl-2 is the typical antiapoptotic protein in the mitochondrial membrane, while Bak is expressed in the cytosol. When apoptosis occurs, Bak is translocated into the mitochondrial membrane and induces mitochondrial dysfunction and subsequently cytochrome c is released from mitochondria into cytoplasm [35]. Along with the Bcl-2 family, the caspase family are regarded as regulators of apoptosis. Caspases are categorized into initiators, including caspase-2, -8, and -9, and effectors, including caspase-3, -6, and -7 [36]. In this study, mitochondrial membrane potential loss, Bcl-2 down-regulation and Bak upregulation, cytochrome c, which promotes formation of apoptosome translocation, and caspase activation are detected by treating *α*-mangostin, suggesting that *α*-mangostin-induced apoptosis is related to the mitochondrial pathway and the caspase cascade.

EMT is important for tumor invasion and the metastasis process [37, 38]. During EMT, primary site epithelial cells undergo morphological and genetic alterations that take on a mesenchymal character, resulting in secondary tumor formation at another site [39, 40]. Thus, controlling of EMT is considered a promising approach to the inhibition of metastasis. In the present study, we found that *α*-mangostin reduced MG-63 cell migration and invasion capability through regulation of EMT-related marker protein expressions. E-cadherin expression, which is an epithelial marker, increased while N-cadherin expression, which is a mesenchymal marker, decreased dose-dependently. Transcription factors, slug and snail protein, and mRNA expressions also decreased after being treated with *α*-mangostin. The MAPK pathway is upstream intracellular signaling and regulates the various cellular processes, including apoptosis and cell migration and invasion [41, 42]. Chen et al. reported that *α*-mangostin suppressed renal carcinoma cell metastasis by ERK expression [43]. Expressions of MAPK family proteins (ERK1/2, JNK, and p38) in this study and phosphorylation of ERK1/2 and p38 decreased in a time-dependent manner. JNK phosphorylation expression was not detected by western blot. Our results suggest that *α*-mangostin induced apoptosis through the mitochondrial pathway and reduced cell migration and invasion through EMT inhibition. Also, *α*-mangostin stimulated MAPK pathway activation in human osteosarcoma MG-63 cells.

This study has several limitations as the experimental concentrations of *α*-mangostin used in* in vitro *data may not be confirmed through an* in vivo* study. In addition, it is difficult to investigate the cytotoxic effect in various normal cells. *α*-Mangostin-stimulated MAPK signaling action was not confirmed by MAPK inhibitors during the apoptosis and EMT inhibition processes.

## 5. Conclusions

The present study demonstrated the molecular mechanisms underlying the chemotherapeutic properties of *α*-mangostin on human osteosarcoma MG-63 cells and showed the valuable data for the development of a novel anticancer agent.
